# A Recurrent Schwannoma in the Left Distal Ulnar Nerve

**DOI:** 10.7759/cureus.64535

**Published:** 2024-07-14

**Authors:** Logan M Morrey, Sumit Patel, Mayron Lichterman

**Affiliations:** 1 Medicine, Western Michigan University Homer Stryker M.D. School of Medicine, Kalamazoo, USA; 2 Orthopedic Surgery, Western Michigan University Homer Stryker M.D. School of Medicine, Kalamazoo, USA

**Keywords:** schwannomatosis, mass resection, ulnar nerve neuropathy, ulnar nerve, peripheral schwannoma

## Abstract

While peripheral nerve schwannomas have a relatively low incidence, schwannomatosis, the condition in which one forms multiple recurring schwannomas, is an even rarer phenomenon and can be hard to detect given its ability to mimic other conditions. We report a case of a 35-year-old male who presented with a mass in his left wrist and forearm, volar pain in his forearm, and numbness in his fingers. Magnetic resonance imaging (MRI) revealed a bilobed heterogeneous neural sheath tumor in the distal left ulnar nerve. The tumor was resected including extensive internal neurolysis using a Zeiss operative microscope. Post-operative biopsy confirmed an encapsulated schwannoma. The patient did well initially but developed worsening pain in his forearm and weakness. He had persistent paresthesias in the ulnar nerve distribution. He underwent a repeat MRI almost one year later, which showed thickening of the ulnar nerve proximal to the area of resection with an 8.5 mm hyperintense nodule. The patient underwent a subsequent resection with extensive neurolysis, which confirmed that the mass was a benign non-invasive schwannoma. At six weeks post-surgery, the patient’s forearm pain was significantly improved and his range of motion returned to baseline. Our case demonstrates the importance of post-operative follow-up in schwannomas with appropriate imaging if symptoms persist or recur.

## Introduction

Although schwannomas are the most common peripheral nerve tumor, comprising 90% of peripheral nerve tumors, they have a relatively low incidence of 0.6 in 100000 [1,2]. These tumors are more prevalent in the upper extremities with studies showing rates as high as 19% of tumors in the upper extremities [3]. Symptoms of peripheral schwannomas are similar to those of other peripheral neurologic pathologies, such as a palpable mass along the peripheral nerve, loss of nerve function presenting as numbness or muscle weakness, and/or pain [4]. Due to their rarity and symptom presentations, schwannomas can be mistaken for ganglion cysts or carpal tunnel syndrome [5]. Schwannomas are relatively benign and slow-growing. Additionally, they are encapsulated, making them unlikely to permanently damage nerve bundles. The mainstay of treatment is surgical excision with marginal resection. Their non-invasive nature makes this a relatively benign procedure as there is no need to remove the associated nerves or nerve bundle [6].

The presence of multiple schwannomas in a single nerve, or schwannomatosis, has an even rarer occurrence and to date has only been described in case reports [7-9]. Treatment of schwannomatosis should depend on the size of recurring masses and symptoms. Occasionally, patients with schwannomatosis will be symptom-free having no gross remnant masses after initial excision with the only evidence for recurring tumor found on imaging. In these cases, a surgical team can elect to conservatively manage the patient with yearly follow-up and further imaging if symptoms recur [7].

We report a case of an ulnar nerve schwannoma in a 35-year-old male with five years of symptoms before initial resection and a recurring schwannoma one year after the index procedure.

## Case presentation

A 35-year-old male first presented with atypical carpal tunnel syndrome, with paresthesia involving all five fingers in the left hand in late 2016. His symptoms did improve minimally with a diagnostic carpal tunnel injection. The patient underwent left carpal tunnel release (CTR) in early 2017 with improvement in his symptoms but had persistent tingling in his fingers and palmar pain near his metacarpal heads. The patient began to develop a shooting volar forearm pain with associated thumb numbness and extreme cold sensitivity two months post-op. At a five-month follow-up, his pain worsened and the patient also developed wrist flexion weakness. He wore a left wrist brace and was placed on work restrictions due to his symptoms. He was subsequently lost to follow-up.

**Figure 1:**
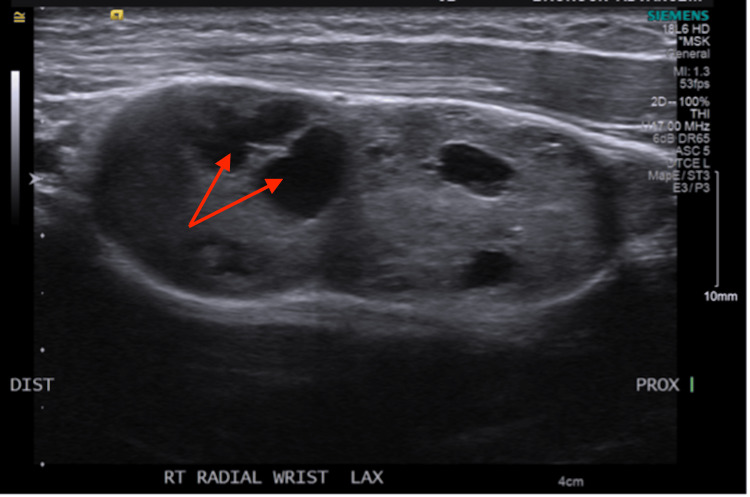
Ultrasound image showing a bilobed dumbbell-shaped mass in the distal left forearm. The mass is centered within the soft tissues, underlying the adjacent flexor tendons, and measures 2.8x1.4x2.0 cm proximally and 5.4x1.9x2.67 cm more distally. MRI, magnetic resonance imaging

**Figure 2:**
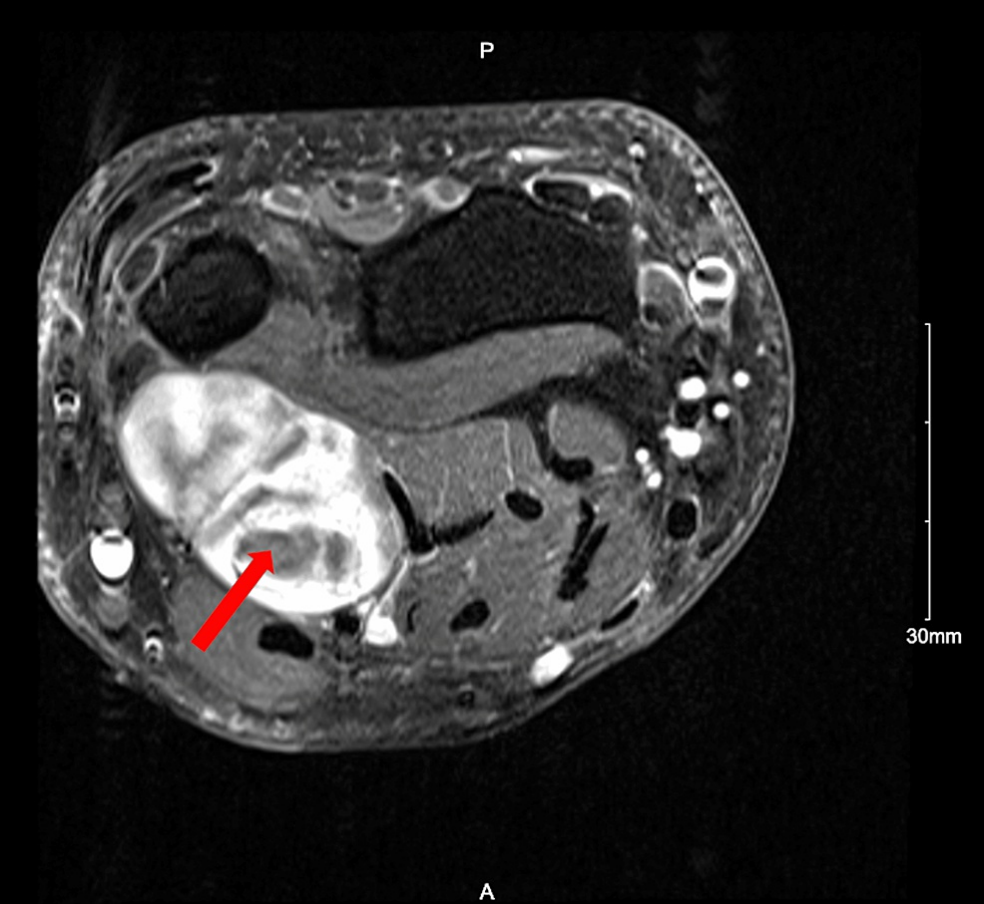
Axial T2 MRI of the left forearm showing the bilobed neural sheath tumor of the ulnar nerve in the distal 1/3 of the forearm. The mass is positioned between the flexor carpi ulnaris muscle and the flexor digitorum profundus and to a lesser extent flexor digitorum superficialis muscles. The mass terminates at the distal ulna with no extension into the region of Guyon's canal. Contrast administration showed heterogeneous consistency with areas of probable cystic change or internal necrosis. MRI, magnetic resonance imaging

The patient re-presented in 2022 complaining of numbness and tingling in his fingers as well as a mass in his left wrist and forearm. Physical examination showed a 4 cm mobile, poorly circumscribed mass in the volar/ulnar wrist and distal forearm. He had worse numbness and tingling in his fingers with pressure over the mass and a positive Tinel test over the mass. Ultrasound of the left distal ulnar forearm showed a dumbbell-shaped mass with a few separate components measuring up to 5.4 cm in proximal dimensions and 2.8 cm in distal dimensions most likely related to a nerve sheath tumor (Figure 1). Magnetic resonance imaging (MRI) suggested the mass was either two separate or a bilobed neural sheath tumor that was heterogeneous, likely due to cystic degeneration (Figure 2). Ultrasound and MRI imaging found no other nerves were affected.

The patient underwent resection of the encapsulated tumor through extensive internal neurolysis under a Zeiss operative microscope and Guyon’s canal release in September 2022 with no complications. Intraoperatively the mass appeared multiloculated and contained three large components that appeared to be connected measuring a total of 7 cm in length. Fibroareolar attachments connecting the mass to surrounding tissues were separated using tenotomy scissors. Individual fascicles of the ulnar nerve were wrapped around the fibrous capsule of the mass, which caused the fascicles to be stretched and distorted. The fascicles were mobilized from the mass using a Zeiss operative microscope. The normal anatomy of the ulnar nerve was severely distorted. There were three fascicles coursed into and were unable to be differentiated from the mass that was sacrificed to remove the mass in its entirety. The cut ends of the nerve were reattached along by the epineurium. Additionally, it was unable to be determined if several fibrous bands running longitudinally were stretched nerve fascicles, and as such these were also sacrificed. The tumor was well encapsulated and removed in three pieces. A biopsy confirmed the mass to be a non-malignant schwannoma.

**Figure 3:**
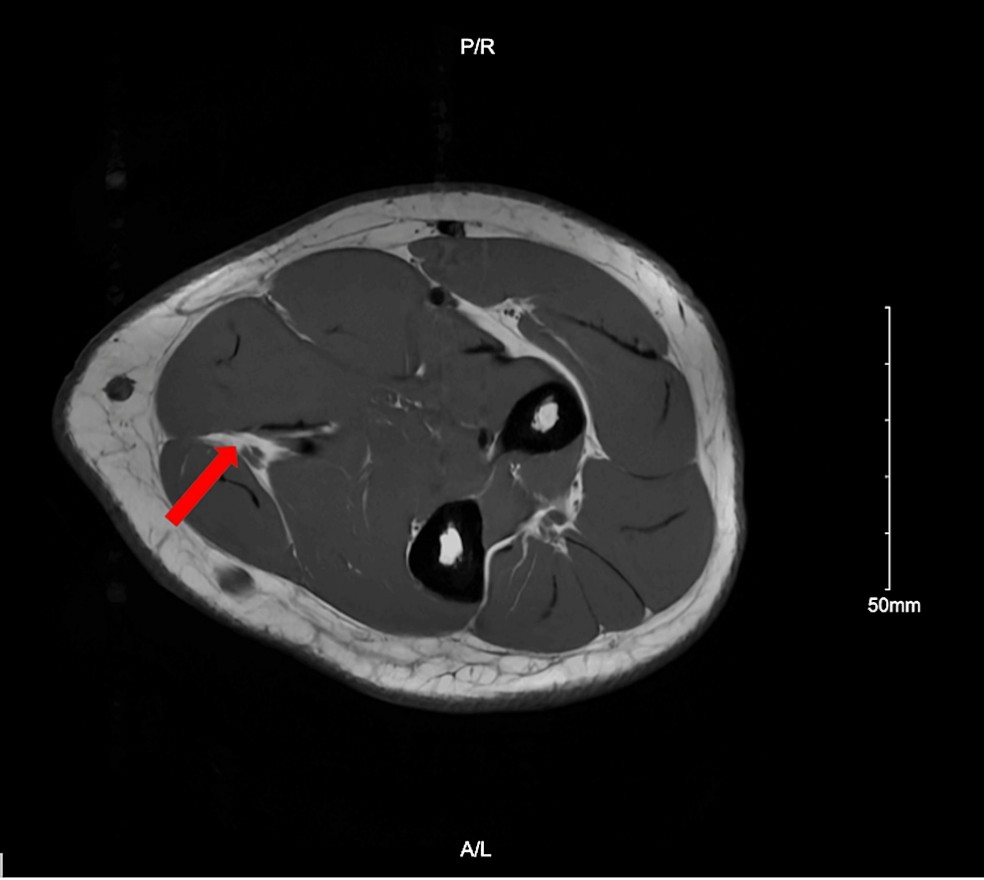
Axial T2 inversion recovery MRI of the left distal 1/3 of the forearm showing nerve sheath tumor, which measures about 5 mm oblique transverse by 8.5 mm proximal-distal by 4 mm. The tumor is within an 8.5 cm segment of the thickened ulnar nerve along the previous resection site.

Post-operatively, the patient experienced persistent numbness in his left ring and small fingers with no improvement in the small finger. He had some improvement in his ring finger initially, which then worsened. He began to develop intermittent paresthesia in his long finger and significant forearm pain, which was worse distally. He was taking high-dose gabapentin three times daily but still had pain. His scar was tender to palpation and hypertrophic. He had regained full wrist and finger motion following surgery. MRI of the forearm confirmed thickening of the neural sheath with hyperintense enhancing nodule proximal to the area of resection measuring about 5 mm by 8.5 by 4 mm (Figure 3). Electromyography (EMG) confirmed left ulnar mononeuropathy. The mass was once again restricted to the left ulnar nerve; no other nerves were affected.

The patient underwent a subsequent resection in November 2023 with extensive neurolysis of the ulnar nerve with nerve wrap application and a Guyon’s canal release. The ulnar nerve was mobilized using tenotomy scissors and was encased in significant scar tissue from the previous surgery. A small palpable and visible mass was just proximal to the scarring of the ulnar nerve. Ulnar nerve fascicles were dissected using an operative microscope until a well-circumscribed, non-invasive mass was separated consistent with schwannoma. The surrounding fascicles were normal and unaffected by the mass. Pathology confirmed that the repeat tumor was a benign non-invasive schwannoma. 

Although initially reporting worsened numbness of the long, ring, and small fingers compared to preoperation, the patient had a good range of motion of the wrist and fingers. His post-operative pain was significantly improved six weeks after the procedure, and he began to wean the gabapentin.

## Discussion

This case highlights the importance of considering schwannomas in the differential diagnosis of upper extremity masses. Schwannomas can be missed due to their rarity and likeness in presentation to ganglion cysts, carpal/cubital tunnel syndrome, and neurofibromas [4,10]. Our patient likely had an ulnar nerve schwannoma five years before the initial resection when he underwent his CTR. Although his median nerve symptoms were relieved by the CTR, the ring and small finger involvement and his volar forearm pain make this a strong possibility. The patient worked as a butcher prior to his CTR. His post-operative work restrictions along with the slow-growing nature of schwannomas likely masked his symptoms over the course of five years. 

Despite their rarity, schwannomas can be differentiated from other forearm pathologies by several distinct exam, imaging, and pathology findings. Due to compression of the nerve on palpation, other case reports have described schwannomas as tender [11,12]. Their masses are also mobile, and most have a positive Tinel’s sign; all findings are present in our patient [10,13]. An MRI finding highly correlated with schwannomas is the presence of a “target sign,” which describes the mass as having a hypointense center with a surrounding area of hyperintensity and can be seen in our patient in both the initial and recurring masses (Figures 2, 3) [10]. A systematic review of 199 patients with schwannoma showed 59% displayed target signs with a specificity of 100% [14]. Neurofibromas, another benign neural sheath tumor, share these same physical exam characteristics as well as displaying the target sign [15]. Schwannomas can be distinguished for Neurofibromas through pathology as schwannomas are encapsulated and stain positive for S100 protein, which was the case for our patient on the pathology report [10].

Another occurrence for clinicians to be aware of is the possibility of, albeit even rarer, schwannomatosis. This case illustrates the importance of regular follow-up after excision of peripheral nerve tumors. In one case similar to ours, two additional schwannomas were found proximal to the tumor resected on the ulnar nerve, but the patient’s symptoms were limited to intermittent numbness post-operatively. The treatment team elected to manage these proximal tumors conservatively due to minimal symptoms and risks of iatrogenic ulnar nerve injury [16]. In contrast, we elected to resect the second tumor because the benefits of significant pain and symptom relief outweighed the risks. Although surgical excision may be unnecessary in asymptomatic cases, the slow growth of schwannomas and the possibility of recurrence make regular follow-up tantamount [7]. Additionally, schwannomas can affect deep nerves or be small, making them nonpalpable on physical exam, which makes imaging an essential piece of follow-up in symptomatic patients [17]. Treatment of schwannomatosis should depend on the size of recurring cysts and symptoms.

## Conclusions

Schwannomas are benign neural sheath tumors that are rare, especially in peripheral nerves. Because of their rarity, ability to mimic other common upper extremity pathologies, and occasionally being non-palpable, schwannomas and schwannomatosis can be easily overlooked. Our case demonstrates the importance of history, physical exam, imaging, and pathology in diagnosing and treating schwannomas. Distinct features of schwannomas in these entities are tenderness to palpation, mobility, positive Tinel’s sign, MRI target sign, and positive S100 staining. Although rare, schwannomatosis must be considered in the setting of post-operative care in schwannomas and necessitates regular and consistent follow-up. Imaging may be appropriate if there is a persistence of neurological symptoms such as shooting or burning pains, weakness, or numbness. If imaging shows an additional mass and the patients are symptomatic enough that the benefits outweigh the risks, surgical management may be indicated.
